# Detection of Zika Virus in Semen

**DOI:** 10.3201/eid2205.160107

**Published:** 2016-05

**Authors:** Barry Atkinson, Pasco Hearn, Babak Afrough, Sarah Lumley, Daniel Carter, Emma J. Aarons, Andrew J. Simpson, Timothy J. Brooks, Roger Hewson

**Affiliations:** Public Health England, Porton Down, UK (B. Atkinson, P. Hearn, B. Afrough, S. Lumley, D. Carter, E.J. Aarons, A.J. Simpson, T.J. Brooks, R. Hewson);; National Institute for Health Research, Liverpool, UK (T.J. Brooks, R. Hewson)

**Keywords:** Zika, diagnostic, semen, flaviviruses, viruses, sexual transmission

**To the Editor:** As an increasing number of autochthonous Zika virus infections are reported from several South America countries (*1*), we read with interest the report from Musso et al. on the potential sexual transmission of Zika virus (*2*). We report additional evidence for this potential route of transmission after identification of an imported case of infection into the United Kingdom.

After an outbreak alert for Zika in French Polynesia, active screening was implemented at Public Health England (Porton Down, United Kingdom). In 2014, a 68-year-old man had onset of fever, marked lethargy, and an erythematous rash 1 week after returning from the Cook Islands. Serum samples taken 3 days into the febrile illness tested negative for dengue and chikungunya viruses by real-time reverse transcription PCR (rRT-PCR). Test results for dengue virus IgM and chikungunya virus IgM also were negative; a test result for dengue virus IgG was indeterminate.

An rRT-PCR test result for Zika virus (*3*) was positive and indicated a crossing threshold value of 35 cycles. This low viral load, commonly observed even in the acute phase of disease (*3*), meant that attempts to obtain sequence data were unsuccessful. Convalescent-phase serum, urine, and semen samples were requested; only semen was positive for Zika virus by rRT-PCR, at 27 and 62 days after onset of febrile illness. These results demonstrated stronger signals than those obtained in tests of the original serum sample, with crossing threshold values of 29 and 33 cycles, respectively. Zika virus–specific plaque reduction neutralization test results were positive on convalescent-phase serum samples.

Although we did not culture infectious virus from semen, our data may indicate prolonged presence of virus in semen, which in turn could indicate a prolonged potential for sexual transmission of this flavivirus. Moreover, these findings could inform decisions regarding what control methods are implemented and which specimen types are best suited for diagnostic detection.
